# Factors Associated with Suspected Scoliosis Identified Through School Screening: The Role of Body Mass Index and Sports Participation

**DOI:** 10.3390/healthcare14121672

**Published:** 2026-06-12

**Authors:** Josipa Glavaš, Roberta Matković, Mirjana Rumboldt, Jure Aljinović

**Affiliations:** 1 Department of School and Adolescent Medicine, Teaching Institute for Public Health, Split-Dalmatia County, 21000 Split, Croatia; 2 Department of Mental Health, Teaching Institute for Public Health, Split-Dalmatia County, 21000 Split, Croatia; roberta.matkovic@nzjz-split.hr; 3 Department of Family Medicine, University of Split School of Medicine, 21000 Split, Croatia; mirjana.rumboldt@mefst.hr; 4 Institute of Physical Medicine and Rehabilitation with Rheumatology, University Hospital Split, 21000 Split, Croatia; jure.aljinovic@mefst.hr; 5 School of Medicine, University of Split, 21000 Split, Croatia; 6 Faculty of Health Sciences, University of Split, 21000 Split, Croatia

**Keywords:** scoliosis screening, suspected scoliosis, body mass index, sports participation, forward bend test, adolescents

## Abstract

**Background/Objectives**: Adolescent idiopathic scoliosis (AIS) is a multifactorial condition with an unclear etiology. Previous studies have reported associations of scoliosis with female sex, lower body mass index (BMI), and lower physical activity levels. This study examined factors associated with suspected scoliosis identified through school screening, with emphasis on BMI and sports participation. **Methods**: A cross-sectional study was conducted during the 2019/2020 school year and included 18,216 adolescents. Suspected scoliosis was identified using the forward bend test (FBT). Logistic regression analysis assessed associations between suspected scoliosis and sex, grade, BMI, participation in the seven most frequently reported sports, and training frequency. **Results**: Higher BMI (odds ratio (OR) = 0.91, *p* < 0.001), participation in soccer (OR = 0.64, *p* < 0.001), gymnastics (OR = 0.58, *p* = 0.05), martial arts (OR = 0.66, *p* = 0.02), and higher recreational training frequency (OR = 0.92, *p* < 0.001) were associated with lower odds of suspected scoliosis. Female sex (OR = 2.49, *p* < 0.001) and higher grade level (6th: OR = 1.54; 8th: OR = 2.98; *p* < 0.001) were associated with increased odds of suspected scoliosis. **Conclusions**: Suspected scoliosis identified through school screening was more frequently observed among females and adolescents with lower BMI. Participation in certain sports and greater recreational physical activity were associated with lower prevalence and odds of suspected scoliosis. These findings reflect screening-based associations and do not imply causal relationships. The results support the importance of school-based screening and consideration of body composition and physical activity patterns in the early identification of adolescents with suspected scoliosis.

## 1. Introduction

Regular physical activity (PA) during childhood and adolescence is considered fundamental to healthy growth and development, offering significant physical, psychological, cognitive, and social benefits [1]. However, global surveillance data demonstrate that most children and adolescents fail to achieve recommended PA levels [2,3,4]. This contributes to a greater burden of chronic disease and other adverse health outcomes in later life [1,4].

One condition of increasing interest in this context is adolescent idiopathic scoliosis (AIS), a three-dimensional spinal structural deformity characterized by lateral curvature and vertebral rotation. It typically manifests during the pubertal growth spurt [5,6,7,8,9]. AIS predominantly affects girls, with prevalence rates approximately twice those observed in boys [5,8,9]. Beyond spinal deformity, AIS may alter body shape and posture, contribute to back pain, and, in severe cases, impair cardiopulmonary function [9,10].

Despite extensive investigation, the etiology of AIS remains elusive and is considered multifactorial. Proposed mechanisms include genetic predisposition, rapid pubertal growth, hormonal and metabolic factors, neuromuscular and proprioceptive dysfunction, connective tissue abnormalities, and environmental and lifestyle influences [7,9,11,12]. Recent systematic reviews suggest that altered proprioception, impaired neuromuscular control, and deficits in postural stabilization may contribute to abnormal spinal alignment and asymmetrical loading during growth and development [13,14]. Proprioception is fundamental to trunk stability, balance, and postural control. Adolescents with AIS appear to exhibit impaired proprioceptive function and greater postural instability, particularly when visual or proprioceptive input is altered [13,14].

From a biomechanical perspective, AIS has been associated with asymmetric neuromuscular loading of the spine and trunk musculature, which may contribute to altered spinal growth [11,14]. Paramento et al. [14] reported greater activation of the intrinsic spinal muscles on the convex side of the curve in adolescents with AIS. According to Smit et al. [11], reduced muscular loading and lower muscle mass may decrease spinal compression, allowing relatively greater vertebral and intervertebral disc growth during the pubertal growth spurt. Similarly, Barrios et al. [15] reported progressive reductions in BMI and changes in body composition (somatotype) among girls with AIS. These findings suggest that altered neuromuscular control, asymmetric spinal loading, and reduced muscular loading may contribute to AIS pathogenesis. Consistent with these biomechanical hypotheses, adolescents with AIS are often characterized by distinct anthropometric features. Compared with their peers, they tend to be taller and lighter, with lower BMI, reduced systemic bone mass, and lower bone mineral density [12,15,16,17].

Physical activity may be particularly relevant in this context, as it has been shown to improve bone health, muscular strength, balance, and neuromotor development in children and adolescents [12]. Since AIS develops during a period of rapid somatic growth and neuromuscular adaptation, PA has been hypothesized to influence the development and progression of spinal deformity [7]. However, findings remain inconsistent. Some studies report no association between PA and AIS [12], while others report either higher or lower PA levels among adolescents with scoliosis [12,18,19,20]. Nevertheless, emerging evidence suggests that lower levels of physical activity may be associated with a higher likelihood of AIS. In contrast, participation in organized sports does not appear to be harmful and may be linked to a lower likelihood of AIS [9,19,21]. Even current scoliosis-specific rehabilitation approaches generally encourage continued participation in physical activity and sports [22]. The Lyon approach considers sports an integral component of treatment and favors activities that promote symmetrical loading, balance, postural control, and bone health [22]. Conversely, the Scientific Exercise Approach to Scoliosis (SEAS) and the Barcelona Scoliosis Physical Therapy School (BSPTS) emphasize maintaining participation in sports that align with the adolescent’s interests and preferences [22]. Overall, these rehabilitation approaches support an active lifestyle rather than restricting sports participation in adolescents with scoliosis.

The relationship between physical activity and AIS should be considered alongside individual characteristics. Female sex and lower BMI have consistently been identified as factors associated with scoliosis, and these may interact with lifestyle factors such as physical activity and sports participation [15,16,19,23]. Participation in sports is not randomly distributed across the adolescent population. Still, it is often influenced by sex, body composition, somatotype, and biological maturation, with athletes frequently self-selecting or being guided toward disciplines that match their physical characteristics [24,25,26,27,28]. For example, sports such as gymnastics, dance, and volleyball often attract adolescents with leaner, more flexible builds, whereas soccer and combat sports are generally associated with greater muscularity and more robust physical characteristics [26,27,28,29,30,31,32]. Therefore, understanding AIS requires an integrated approach that considers both biological and behavioral factors, as well as the characteristics that may influence participation in specific sports.

Given the global emphasis on increasing physical activity in youth and the ongoing uncertainty regarding its role in AIS, the present study aimed to examine factors associated with suspected scoliosis in a large adolescent population, with particular emphasis on body mass index and sports participation. Based on previous evidence, we hypothesized that suspected scoliosis identified through school screening would be more frequently observed among female adolescents and those with lower BMI. We further hypothesized that participation in organized sports and higher levels of physical activity would be associated with lower odds of suspected scoliosis.

## 2. Materials and Methods

### 2.1. Study Design and Participants

The study was approved by the Ethical Committee of the University of Split School of Medicine (Class: 003-08/22-03/0003; Registry number: 2181-198-03-04-22-0068) and the Ethical Committee of the Teaching Institute for Public Health, Split-Dalmatia County (Class: 500-01/19-01/7; Registry number: 2181-103-01-19-1). A cross-sectional study was conducted during the 2019–2020 school year. The study population comprised students in the 5th, 6th, and 8th grades from four Croatian counties, corresponding approximately to ages 11–14 years in the Croatian educational system. Typical ages were 10–11 years in 5th grade, 11–12 years in 6th grade, and 13–14 years in 8th grade. Written informed consent was obtained from parents, and assent was obtained from the children.

Inclusion criteria were elementary school pupils in the 5th, 6th, and 8th grades who underwent regular school-based preventive examinations and whose parents provided written informed consent. Exclusion criteria included refusal of consent, incomplete survey forms, missing data required for the analyses, and scoliosis associated with congenital, inflammatory, traumatic, neuromuscular, or other known conditions.

The target population comprised 34,530 pupils attending the 5th, 6th, and 8th grades in four Croatian counties. Due to disruptions caused by the COVID-19 pandemic, 7169 pupils were not examined. Of the 27,361 examined pupils, participation was declined by parents or pupils in 8619 cases. Consequently, 18,742 pupils were enrolled in the study. Of the 18,742 participants initially enrolled, 526 were excluded because of incomplete or invalid survey responses (missing data). The proportion of excluded participants due to missing data was low (526/18,742; 2.8%). Consequently, a complete case analysis was performed, and the final analytical sample comprised 18,216 adolescents. This represented 66.6% of the examined population and 52.8% of the target population.

An a priori sample size calculation was not performed because the study included all available participants from the school-based screening program who met the eligibility criteria during the study period. Although no formal power calculation was conducted, the large sample size (*N* = 18,216) was considered sufficient to support the planned multivariable analyses and to detect small associations.

### 2.2. Data Collection and Measures

Data were obtained using structured survey forms specifically developed for this study, incorporating information from medical records (including age, sex, school grade, family history of scoliosis, physical activity, and sedentary behavior), anthropometric measurements, and clinical indicators of scoliosis. The dataset also included records of clinical assessments and follow-up procedures performed by school medicine specialists.

Screening for scoliosis was performed using the forward bend test (FBT), originally described by Adams [33]. The FBT is routinely used in school medicine practice in Croatia and is performed by trained school medicine specialists as a practical, low-cost, and widely implemented screening tool for scoliosis, with reported sensitivity, specificity, and predictive values of approximately 90% [18,19]. In Croatia, school medicine specialists are responsible for the preventive healthcare of schoolchildren within a nationally organized system that ensures universal coverage. Scoliosis screening is part of mandatory preventive examinations conducted during key periods of growth and development and follows a two-step pathway. School medicine specialists perform the initial screening examination using FBT, while children with suspected scoliosis are referred for further evaluation by orthopedic or physical medicine specialists when clinically indicated. Because the FBT is a screening rather than a diagnostic method, pupils with a positive FBT were classified as having suspected scoliosis identified through school screening. In contrast, those with a negative FBT were classified as non-suspected scoliosis. Since follow-up diagnostic assessments were not uniformly completed and documented for all screened participants, the present study focused on suspected scoliosis identified during school screening rather than on radiographically confirmed AIS.

Anthropometric measurements were obtained by school nurses using standardized procedures to measure height and body weight. Body mass index (BMI) was subsequently calculated and categorized according to age- and sex-specific BMI-for-age percentiles [34].

Physical activity was assessed based on participation in organized sports and recreational physical activity, including training frequency and duration (number of weekly sessions). Organized sport was defined as structured physical activity conducted under the supervision of a coach or trainer, with scheduled training sessions and formal practice. In contrast, recreational physical activity was classified as irregular because it does not necessarily meet standardized criteria for frequency, duration, or intensity. Participation in the seven most frequently represented sports included in the primary analyses (soccer, gymnastics, martial arts, volleyball, handball, basketball, and dance) was recorded, together with an “other sports” category and a “no sports participation” category. The “other sports” category comprised all remaining sports reported by participants, including swimming, athletics, tennis, rowing, and several less-represented disciplines. Because a total of 31 sports were reported, only the most frequently represented sports, with sufficient numbers and relevance to the study objectives, were analyzed separately. The remaining sports were grouped into the “other sports” category to ensure adequate group sizes and interpretable comparisons.

### 2.3. Statistical Analysis

All statistical analyses were performed using IBM SPSS Statistics version 20.0 (IBM Corp., Armonk, NY, USA). Descriptive statistics are presented as frequencies and percentages.

To examine factors associated with suspected scoliosis, logistic regression analysis was conducted. The model included sex, grade, body mass index (BMI), participation in the seven most frequently reported sports, and the frequency of active and recreational training sessions as independent variables. Results are presented as odds ratios (OR) with 95% confidence intervals (CI).

Suspected scoliosis was coded as the dependent binary outcome variable (0 = no, 1 = yes). Sex was coded as a binary variable (1 = male, 2 = female), while participation in individual sports was coded dichotomously (0 = no, 1 = yes). Grade level was entered as a categorical variable, with 5th grade used as the reference category. BMI and the number of weekly active and recreational training sessions were analyzed as continuous variables. Reference categories in the regression model were male sex, 5th grade, and no participation in the respective sport.

All analyses were performed after verification of the underlying assumptions. Multicollinearity was assessed using variance inflation factors (VIFs), and model fit was evaluated using the Hosmer–Lemeshow and Omnibus tests. Model performance was assessed using Nagelkerke R^2^, classification accuracy, and receiver operating characteristic (ROC) analysis, including the area under the curve (AUC), sensitivity, and specificity. A *p*-value < 0.05 was considered statistically significant.

## 3. Results

The total study sample comprised 18,216 adolescents (9131 males and 9085 females) from the 5th to 8th grades, corresponding approximately to ages 11–14 years. Physical activity was assessed through participation in organized sports and recreational physical activity, as well as the number of weekly training sessions. In descriptive analyses, 49.6% of students participated in organized sports training, 27.0% reported recreational physical activity, and 23.5% reported no regular physical activity. Among students participating in organized sports, the most common frequency was three training sessions per week (42.4%), whereas among those engaged in recreational physical activity, it was two sessions per week (32.5%).

The mean body mass index (BMI) was 20.3 kg/m^2^. BMI was analyzed as a continuous variable in the regression model and categorized for descriptive purposes.

Table 1 presents the prevalence of suspected scoliosis identified through school screening according to sports participation and BMI category. The prevalence of suspected scoliosis was lowest among adolescents participating in soccer (2.8%), martial arts (3.9%), and gymnastics (4.7%). Higher prevalences were observed among volleyball players (8.2%), dancers (7.7%), and adolescents not participating in sports (6.7%). Across all sports categories, the majority of participants did not have suspected scoliosis, with proportions ranging from 91.8% to 97.2%.

Among adolescents with suspected scoliosis, most cases occurred in the underweight and normal-weight categories, whereas relatively few cases were observed among overweight and obese participants. Similar patterns were observed across the different sports disciplines and among adolescents who did not participate in organized sports. These findings are further illustrated in Figure S1 (Supplementary Material).

To examine the contribution of sociodemographic variables (sex, age, and BMI), participation in one of the seven most common sports, and level of physical activity (active and recreational training) in predicting suspected scoliosis, a logistic regression analysis was performed on the full sample. BMI was entered into the model as a continuous variable, whereas body weight, body height, and BMI categories were excluded from the final model to avoid redundancy, reduce multicollinearity, and preserve statistical power, as categorizing continuous variables can reduce power and weaken observed associations. Before the analysis, the assumptions underlying logistic regression were assessed and found to be adequately met. The sample size was adequate, and variance inflation factors (VIFs) were all below 5, indicating no multicollinearity. The Hosmer–Lemeshow test indicated good model fit (χ^2^(8) = 10.373, *p* > 0.05), while the Omnibus test confirmed that the model significantly improved fit compared with the null model (χ^2^(13) = 526.709, *p* < 0.001). The model explained 8% of the variance in suspected scoliosis (Nagelkerke R^2^ = 0.08) and achieved an overall classification accuracy of 94.2%. To further evaluate model performance, receiver operating characteristic (ROC) analysis was performed. The area under the curve (AUC) was 0.705 (SE = 0.008; 95% CI: 0.690–0.720; *p* < 0.001), indicating acceptable but modest discriminatory ability. However, at the default classification threshold of 0.500, the model classified all participants as non-suspected scoliosis, resulting in a sensitivity of 0.0% and a specificity of 100.0%. Therefore, the high overall classification accuracy primarily reflected the predominance of the non-suspected scoliosis group and the correct classification of this group.

Statistically significant predictors of suspected scoliosis included sex, grade, BMI, participation in soccer, gymnastics, and martial arts, and the number of recreational training sessions (Table 2). In females, higher odds of suspected scoliosis were observed (OR = 2.49, *p* < 0.001), as were in higher grades (6th grade: OR = 1.54; 8th grade: OR = 2.98; both *p* < 0.001). In contrast, higher BMI (OR = 0.91, *p* < 0.001), participation in soccer (OR = 0.64, *p* < 0.001), gymnastics (OR = 0.58, *p* = 0.05), and martial arts (OR = 0.66, *p* = 0.02), as well as a greater number of recreational training sessions (OR = 0.92, *p* < 0.001), were associated with lower odds of suspected scoliosis. None of the other predictors was statistically significant.

## 4. Discussion

### 4.1. Main Findings

The descriptive data showed that most adolescents with suspected scoliosis were in the normal-weight or underweight categories. This pattern was observed across all sports disciplines and among adolescents who did not participate in organized sports. Therefore, it does not appear to be specific to any particular sport. Interpretation of sport-specific findings requires caution. We did not perform formal statistical comparisons between sports disciplines, and several subgroup sizes were relatively small. The observed differences may reflect variations in sex distribution, body composition, flexibility, biological maturation, and sport-selection patterns rather than the effects of the sports themselves.

For example, almost all adolescents with suspected scoliosis participating in volleyball (82 of 83), dance (55 of 55), and gymnastics (14 of 15) were female. In contrast, most soccer players with suspected scoliosis were male (65 of 77). These distributions closely mirror the sex-specific participation patterns of the respective sports. Absolute numbers of suspected scoliosis cases should be interpreted in relation to the size of each sports group. Soccer included 77 adolescents with suspected scoliosis and had the largest number of participants (*n* = 2780). As a result, its prevalence was among the lowest (2.8%). Volleyball included a similar number of suspected scoliosis cases (*n* = 83), despite a much smaller participant group (*n* = 1018). Consequently, its prevalence was substantially higher (8.2%). These findings represent descriptive, screening-based associations. They should not be interpreted as evidence that particular sports are protective against or increase the risk of scoliosis.

A further observation was that the largest absolute number of adolescents with suspected scoliosis was recorded among those who did not participate in organized sports (*n* = 612). The prevalence of suspected scoliosis in non-participants (6.7%) was also higher than the overall prevalence in the study population (5.8%). These observations are consistent with previous reports linking lower levels of physical activity to scoliosis [12,19,20,21,23,35]. However, they should be interpreted with caution because of the cross-sectional design, potential self-selection, and the screening-based nature of the outcome. Further studies using clinically confirmed AIS outcomes are needed to clarify these associations.

More than half of all participants were in the normal-weight category, whereas underweight predominated only among gymnasts. This observation is consistent with previous evidence showing that the relationship between sports participation and BMI in children is generally weak. Organized sports account for only a modest proportion of total daily energy expenditure, and a substantial part of training time may be spent on low-intensity activities [36,37]. Consequently, factors beyond sports participation are likely to play an important role in determining BMI during childhood and adolescence [36,37].

The results of this study indicate that several predictors contribute significantly to school medicine specialists’ suspicion of scoliosis. In particular, sex and school grade emerged as important factors. Female adolescents and those in higher grades showed a greater likelihood of suspected scoliosis. These findings are consistent with previous research demonstrating a higher prevalence of AIS among girls and an increase in occurrence with age, particularly during periods of rapid growth and pubertal development [5,6,9,18,38,39,40]. However, these associations should be interpreted in the context of a screening-based outcome and do not necessarily reflect associations with radiographically confirmed AIS. The observed sex differences are commonly attributed to biological and hormonal factors, including differences in growth velocity, skeletal maturation, and endocrine influences, which may increase susceptibility to spinal deformities during adolescence [7,9].

Lower BMI was associated with higher odds of suspected scoliosis identified through school screening. This finding is consistent with previous studies reporting an association between lower BMI and scoliosis [12,15,16,17,18,23,41]. Similarly, some studies have observed lower scoliosis prevalence among adolescents with higher BMI [42]. However, the magnitude of the association observed in our study was modest (OR = 0.91) and should therefore be interpreted cautiously. Moreover, the descriptive data showed that most adolescents with suspected scoliosis were classified as normal weight or underweight. A similar predominance of normal-weight and underweight adolescents was observed in the overall study population. This suggests that the distribution of suspected scoliosis cases may partly reflect the underlying BMI distribution in adolescents rather than a clinically meaningful BMI effect. Given the cross-sectional design and the screening-based nature of the outcome, these findings do not imply a protective effect of higher BMI. Unmeasured factors may also influence the observed association. These include pubertal maturation, growth patterns, nutritional status, body composition, and other biological characteristics that were not available in the present study.

In the multivariable analysis, participation in soccer, gymnastics, and martial arts was associated with lower odds of suspected scoliosis identified through school screening, whereas no statistically significant associations were observed for the remaining sports. These findings partially align with previous research suggesting that participation in organized sports may be associated with a lower likelihood of scoliosis [19,21,26,43,44,45]. However, these findings should be interpreted with caution because the outcome was based on screening-detected suspected scoliosis rather than radiographically confirmed AIS. The cross-sectional design also precludes causal inference.

The observed associations may reflect self-selection into sports, sport-specific recruitment practices, or underlying differences in body composition, flexibility, and biological maturation. Adolescents with particular physical characteristics may be more likely to choose certain sports or be encouraged by parents and coaches to participate in them [24,25,28,31,32]. Therefore, the lower odds observed in soccer, gymnastics, and martial arts should not be interpreted as evidence of a protective effect of these sports. Rather, they indicate associations observed within a school-screened population that warrant further investigation in prospective studies using clinically confirmed AIS outcomes. Other unmeasured factors, including growth-related characteristics, family predisposition, and neuromuscular or biomechanical traits, may also have contributed to the observed associations.

The overall level of physical activity, including additional recreational training, showed a limited and inconsistent relationship with suspected scoliosis. This finding suggests that the relationship between physical activity and suspected scoliosis may vary according to the type and intensity of activity. The number of recreational training sessions was identified as a significant predictor, whereas the number of active (organized) training sessions was not. This finding suggests that general recreational physical activity may be related to musculoskeletal characteristics, posture, balance, and neuromotor control. Similar observations have been reported in studies highlighting the benefits of moderate, diverse physical activity for postural development and spinal health in children and adolescents [9,12,19,44,45]. However, these findings should be interpreted cautiously. Physical activity was assessed using self-reported training frequency and did not include information on activity intensity, duration, or specific movement patterns. Recreational activities are particularly difficult to characterize because they are often irregular, involve multiple activities, and may vary considerably in intensity even within a single session. Consequently, the observed associations may be influenced by lifestyle and behavioral factors beyond physical activity itself.

Although several variables were statistically associated with suspected scoliosis, the model’s overall explanatory power was modest (Nagelkerke R^2^ = 0.08). This indicates that the included variables accounted for only a small proportion of the variability in suspected scoliosis and that the present model did not capture important determinants. Potentially relevant factors include pubertal maturation, growth-related characteristics, family predisposition, and other biological or biomechanical variables that were not available for analysis. Consequently, the identified predictors should be interpreted as factors associated with suspected scoliosis at the population level rather than as variables with substantial predictive value for identifying affected individuals.

The overall classification accuracy was high (94.2%), but this finding should be interpreted with caution. The study population was characterized by a marked imbalance in outcome distribution, with only 5.8% of participants classified as having suspected scoliosis. As a result, a high overall accuracy may largely reflect the correct classification of the non-suspected scoliosis group. Consistent with this observation, ROC analysis demonstrated only modest discriminatory ability (AUC = 0.705), and the model failed to identify suspected scoliosis cases at the default classification threshold. Taken together, these findings indicate that the model is more useful for identifying statistical associations than for predicting individual cases of suspected scoliosis.

School medicine specialists play an important role in the early identification of suspected scoliosis, ongoing monitoring, and promotion of physical activity among children. They provide counseling on basic corrective exercises for postural abnormalities and encourage participation in sports. Their continuous surveillance of children as they grow enables timely recognition of potential problems, which is particularly relevant given increasingly sedentary lifestyles [1]. Although sport is not a treatment for scoliosis, it contributes to overall physical and cognitive well-being. In line with SOSORT recommendations, regular physical activity is associated with better psychological outcomes and is not considered harmful for individuals with scoliosis [5]. Moreover, observational evidence suggests that more frequent sports participation may be associated with a lower risk of progression in adolescents with idiopathic scoliosis [19,21,23].

Current scoliosis-specific rehabilitation approaches also emphasize maintaining participation in physical activity, with appropriate monitoring and individualized exercise programs when needed [22]. School settings may provide an important opportunity for such initiatives. Existing physical education programs already include activities that support postural development and muscular fitness. However, exercises targeting trunk stabilization, balance, and postural control could be incorporated more systematically. Given that the largest number of adolescents with suspected scoliosis in our sample was observed among those not participating in organized sports, strategies to increase overall physical activity and reduce inactivity may be particularly relevant. School sports facilities may also serve as accessible settings for programs promoting postural health and early intervention for musculoskeletal abnormalities. In Croatia, existing collaborations between schools and community sports organizations could support the implementation of such programs. A multidisciplinary approach involving school physicians, physical education teachers, coaches, physiotherapists, and other healthcare professionals may further improve the early identification, monitoring, and individualized exercise recommendations for adolescents with suspected scoliosis [9,19,24,26].

In this context, the present findings are consistent with the multifactorial nature of AIS and highlight the importance of considering both biological factors, such as sex and BMI, and lifestyle factors, such as sports participation and recreational physical activity. The observed associations should be interpreted within a broader framework that includes biological maturation, body composition, neuromuscular characteristics, and sport selection patterns. These results emphasize the value of targeted school-based screening and continued monitoring, particularly among female adolescents and those with lower BMI. They also support public health strategies to promote regular physical activity and reduce inactivity during adolescence.

### 4.2. Limitations

This study has several important limitations that should be considered when interpreting the findings. First, the cross-sectional study design precludes any inference of causal relationships between the examined factors and suspected scoliosis. Future longitudinal studies are needed to better understand potential causal pathways. In addition, information on pubertal status, growth velocity, family history of scoliosis, nutritional factors, and other biomechanical or neuromuscular characteristics was not available. Consequently, residual confounding cannot be excluded and may have influenced the observed associations.

Second, the model’s overall explanatory power was modest (Nagelkerke R^2^ = 0.08), indicating that only a small proportion of the variance in suspected scoliosis was explained by the included variables. This suggests that the model may not fully capture the complexity of scoliosis development. Consequently, the findings are more informative for understanding population-level associations than for predicting individual-level suspected scoliosis. This limitation underscores the need for future research to incorporate additional biological, developmental, and biomechanical factors that may contribute to scoliosis.

Third, the outcome was based on screening findings (suspected scoliosis) obtained using the forward bend test (FBT), without radiographic confirmation through the referral pathway. While this approach reflects school-based screening practice, it may have led to misclassification of cases due to the FBT’s limited diagnostic accuracy. Future studies that link screening data to clinical follow-up and confirmed diagnoses would yield more robust outcome measures.

Fourth, physical activity was assessed by self-report and did not capture key dimensions such as intensity, duration, or specific movement type. While organized sports typically follow structured schedules with defined duration and frequency, recreational physical activity is often irregular, may involve multiple activities within the same day, and can last for variable periods, making precise quantification difficult. Future research should incorporate objective or more detailed measures of physical activity, including duration (e.g., minutes per session), intensity, and type, to better understand the relationship between physical activity and scoliosis risk.

In addition, the analysis did not include detailed sport-specific variables, and no direct comparisons between different sports disciplines were performed. Future studies should incorporate more detailed sport-related characteristics and apply analytical approaches that enable direct comparisons between disciplines.

## 5. Conclusions

Suspected scoliosis identified through school screening was associated with several biological and constitutional factors, particularly sex, school grade, and body mass index. Female adolescents and those in higher grades were more likely to have suspected scoliosis, whereas higher BMI was associated with lower odds of suspected scoliosis. However, the magnitude of this association was modest, and its interpretation should remain cautious.

Sports participation and physical activity were also associated with suspected scoliosis, although these relationships varied according to the type and context of activity. Participation in soccer, gymnastics, and martial arts was associated with lower odds of suspected scoliosis, whereas no significant associations were observed for the remaining sports. Given the cross-sectional design and the screening-based nature of the outcome, these findings should not be interpreted as evidence of a protective effect of specific sports. Rather, they likely reflect a complex interplay of biological, behavioral, and sport-selection factors.

Despite the statistical significance of several predictors, the model explained only a small proportion of the variance and showed limited ability to identify suspected cases at the individual level. Therefore, the findings should be interpreted as indicative of general patterns rather than precise predictive relationships.

Overall, the findings support the multifactorial nature of AIS and highlight the importance of considering both biological factors and lifestyle-related characteristics when evaluating suspected scoliosis. The results emphasize the value of school-based screening and continued monitoring, particularly among female adolescents and those with lower BMI. They also support efforts to promote regular physical activity and reduce inactivity during adolescence while recognizing that the relationship between physical activity and scoliosis requires further investigation in prospective studies with clinically confirmed AIS outcomes.

## Figures and Tables

**Table 1:** Prevalence of suspected scoliosis identified through school screening according to sports participation and BMI category.

							Suspected Scoliosis		
Sport	Suspected Scoliosis		Non-Suspected Scoliosis		Total	Underweight	Normal Weight	Overweight	Obesity
	*n* (M/F)	(%)	*n* (M/F)	(%)		*n* (%)	*n* (%)	*n* (%)	*n* (%)
Soccer	77 (65/12)	(2.8)	2703 (2490/213)	(97.2)	2780	34 (1.2)	42 (1.5)	1 (0.0)	0 (0.0)
Gymnastics	15 (1/14)	(4.7)	306 (23/283)	(95.3)	321	9 (2.8)	6 (1.9)	0 (0.0)	0 (0.0)
Martial arts	41 (15/26)	(3.9)	1022 (611/411)	(96.1)	1063	11 (1.0)	29 (2.7)	1 (0.1)	0 (0.0)
Volleyball	83 (1/82)	(8.2)	935 (49/886)	(91.8)	1018	37 (3.6)	44 (4.3)	2 (0.2)	0 (0.0)
Handball	28 (14/14)	(3.4)	807 (508/299)	(96.6)	835	9 (1.1)	17 (2.0)	1 (0.1)	1 (0.1)
Basketball	44 (18/26)	(6.7)	615 (428/187)	(93.3)	659	15 (2.3)	28 (4.2)	1 (0.2)	0 (0.0)
Dance	55 (0/55)	(7.7)	658 (36/622)	(92.3)	713	22 (3.1)	32 (4.5)	1 (0.1)	0 (0.0)
Other sports	98 (41/57)	(6.0)	1540 (872/668)	(94.0)	1638	32 (2.0)	61 (3.7)	5 (0.3)	0 (0.0)
No sports participation	612 (140/472)	(6.7)	8577 (3819/4758)	(93.3)	9189	248 (2.7)	321 (3.5)	31 (0.3)	12 (0.1)
Total	1053 (295/758)	(5.8)	17,163 (8836/8327)	(94.2)	18,216	417 (2.3)	580 (3.2)	43 (0.2)	13 (0.1)

Note: Percentages for suspected and non-suspected scoliosis were calculated within each sport category (row percentages).

**Table 2:** Logistic regression analysis of factors associated with suspected scoliosis.

Variable	B	SE	OR	95% CI (Lower)	95% CI (Upper)	*p*
Sex (female)	0.91	0.08	2.49	2.13	2.90	<0.001
Grade (6th)	0.43	0.10	1.54	1.27	1.88	<0.001
Grade (8th)	1.09	0.09	2.98	2.53	3.52	<0.001
BMI	−0.10	0.01	0.91	0.89	0.93	<0.001
Soccer (yes)	−0.45	0.15	0.64	0.48	0.86	<0.001
Gymnastics (yes)	−0.55	0.28	0.58	0.33	1.00	0.05
Martial arts (yes)	−0.42	0.18	0.66	0.46	0.94	0.02
Volleyball (yes)	−0.07	0.15	0.94	0.70	1.24	0.65
Handball (yes)	−0.39	0.22	0.68	0.44	1.04	0.07
Basketball (yes)	0.19	0.18	1.22	0.85	1.74	0.29
Dance (yes)	−0.10	0.16	0.91	0.66	1.25	0.56
Number of active training sessions	0.00	0.03	1.00	0.95	1.05	0.90
Number of recreational training sessions	−0.08	0.02	0.92	0.88	0.96	<0.001

Note: B = unstandardized logistic regression coefficient; SE = standard error; OR = odds ratio; CI = confidence interval; *p* = statistical significance; BMI = body mass index. Reference categories were male sex, 5th grade, and non-participation in the respective sport. Categories shown in parentheses indicate comparisons with the corresponding reference category.

## Data Availability

The data presented in this study are not publicly available due to ethical and privacy restrictions. The dataset includes information collected from minors and contains data derived from medical records. In accordance with ethical approvals and the informed consent provided by participants’ parents/guardians, access to the data is restricted. Data are available from the corresponding author upon reasonable request.

## Supplementary Materials

The following supporting information can be downloaded at: https://www.mdpi.com/article/10.3390/healthcare14121672/s1, Figure S1: Distribution of suspected scoliosis by BMI and sports participation.

## Abbreviations

The following abbreviations are used in this manuscript:

AIS	Adolescent idiopathic scoliosis
BMI	Body mass index
FBT	Forward bend test
PA	Physical activity
