# Supplementary figures and images for: Factors Associated with Suspected Scoliosis Identified Through School Screening: The Role of Body Mass Index and Sports Participation
Source: Healthcare (Basel). 2026 Jun 12;14(12):1672. doi: 10.3390/healthcare14121672 (PMC13299358; doi:10.3390/healthcare14121672)

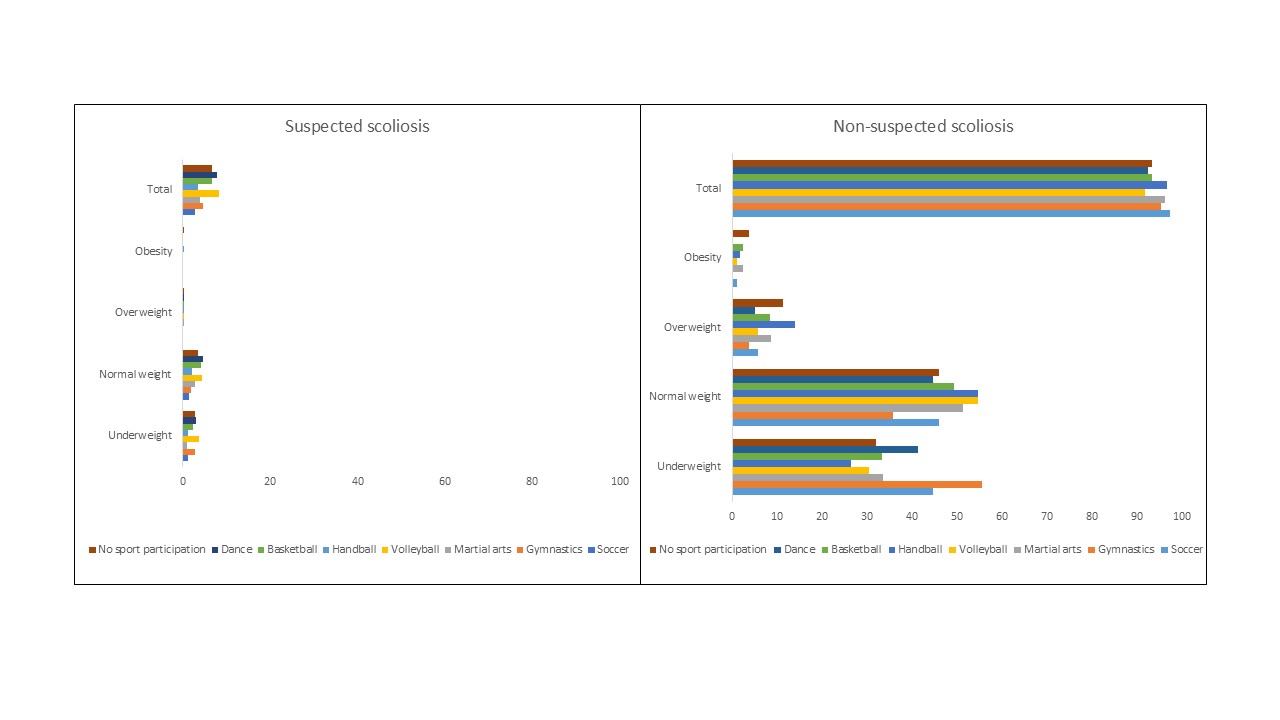

Supplement: Supplementary file 1 [file healthcare-14-01672-s001.zip › healthcare-4310465-supplementary.jpg]
